# The Potential Impact of Large Language Models on Doctor–Patient Communication: A Case Study in Prostate Cancer

**DOI:** 10.3390/healthcare12151548

**Published:** 2024-08-05

**Authors:** Marius Geantă, Daniel Bădescu, Narcis Chirca, Ovidiu Cătălin Nechita, Cosmin George Radu, Stefan Rascu, Daniel Rădăvoi, Cristian Sima, Cristian Toma, Viorel Jinga

**Affiliations:** 1 Department of Urology, “Carol Davila” University of Medicine and Pharmacy, 8 Eroii Sanitari Blvd., 050474 Bucharest, Romania; 2 Center for Innovation in Medicine, 42J Theodor Pallady Bvd., 032266 Bucharest, Romania; 3 United Nations University—Maastricht Economic and Social Research Institute on Innovation and Technology, Boschstraat 24, 6211 AX Maastricht, The Netherlands; 4 Department of Urology, “Prof. Dr. Th. Burghele” Clinical Hospital, 20 Panduri Str., 050659 Bucharest, Romania; 5 Academy of Romanian Scientists, 3 Ilfov, 050085 Bucharest, Romania

**Keywords:** prostate cancer, ChatGPT, Co-Pilot, Gemini, cancer literacy, large language models, doctor-patient relationship

## Abstract

Background: In recent years, the integration of large language models (LLMs) into healthcare has emerged as a revolutionary approach to enhancing doctor–patient communication, particularly in the management of diseases such as prostate cancer. Methods: Our paper evaluated the effectiveness of three prominent LLMs—ChatGPT (3.5), Gemini (Pro), and Co-Pilot (the free version)—against the official Romanian Patient’s Guide on prostate cancer. Employing a randomized and blinded method, our study engaged eight medical professionals to assess the responses of these models based on accuracy, timeliness, comprehensiveness, and user-friendliness. Results: The primary objective was to explore whether LLMs, when operating in Romanian, offer comparable or superior performance to the Patient’s Guide, considering their potential to personalize communication and enhance the informational accessibility for patients. Results indicated that LLMs, particularly ChatGPT, generally provided more accurate and user-friendly information compared to the Guide. Conclusions: The findings suggest a significant potential for LLMs to enhance healthcare communication by providing accurate and accessible information. However, variability in performance across different models underscores the need for tailored implementation strategies. We highlight the importance of integrating LLMs with a nuanced understanding of their capabilities and limitations to optimize their use in clinical settings.

## 1. Introduction

The doctor–patient relationship is a cornerstone of medical practice, tracing its roots back to ancient civilizations like Egypt and Greece, where healers were viewed with a mix of mystical reverence and trust [1]. Hippocrates, often considered the father of modern medicine, formalized this relationship through his ethical principles, emphasizing confidentiality and patient welfare [2]. As medicine progressed through the Middle Ages and into the Renaissance [3], the relationship dynamics shifted with increased emphasis on scientific understanding. However, the fundamental elements of empathy and confidentiality have remained central [4].

The 20th century introduced more complexities with technological advancements and greater patient autonomy, leading to today’s model. During the Industrial Revolution, the doctor–patient relationship underwent significant transformations in response to the profound societal changes brought about by industrialization [5]. The 4th Industrial Revolution, characterized by integrating digital, biological, and physical technologies [6], has further evolved the doctor–patient relationship. Innovations such as telemedicine, electronic health records (EHRs), and AI-driven health technologies have transformed how doctors and patients interact [7]. This era also marked the beginning of professional medical associations and the standardization of medical training and practices, which professionalized the field and altered the dynamic between doctors and patients. This catalyzed a shift from more personal and individualized doctor–patient interaction to a more systematic and formalized approach in medical practice, laying the groundwork for modern medical bureaucracy and clinical practices.

In 1949, Claude Shannon and Warren Weaver introduced the linear communication model, often referred to as the Shannon–Weaver model [8]. This foundational concept was originally developed to enhance telephone communication and later adopted across various fields including health [9]. 

After the public launch of ChatGPT in November 2022, large language models (LLMs) began to be used for various purposes in the healthcare field, from patient information and medical education to simplifying bureaucratic processes in medical institutions and improving the diagnosis and treatment of various diseases [10,11,12]. LLMs such as ChatGPT, Co-Pilot, Gemini, and others are at the forefront of a revolution in technology-enabled health communication. These models utilize deep learning techniques to comprehend and generate human-like language. The core challenge lies in effectively integrating these sophisticated tools into the healthcare system, particularly to enhance doctor–patient communication. The implications of LLMs affecting the doctor–patient relationship have yet to be fully discerned and foreseen. In this landscape, this conversation has recently intensified. Some claim that deploying LLMs will lead to the democratization of medical knowledge, meaning that medical information will become more accessible and understandable to a broader audience beyond the traditional framework [13]. Others deem the use of LLMs as a means to enhance the strategies that healthcare organizations develop to improve the patients’ understanding of medical information [14,15]. 

The integration of LLMs into healthcare presents both challenges and opportunities for the doctor–patient relationship, potentially introducing both *facilitative* and *disruptive elements* to the linear model of doctor–patient communication [16,17,18,19]. Namely, facilitative elements refer to ways in which LLMs can enhance or improve the doctor such as instant access to medical information and advice, tailoring medical advice to the specific needs and history of individual patients, handling routine inquiries and immediate responses to common questions as well as educating and empowering patients. Disruptive elements refer to challenges or negative impacts that LLMs might introduce to the doctor–patient relationship. Specifically, miscommunication (LLMs might not fully understand or appropriately respond to nuanced human emotions or complex medical issues), overreliance (both doctors and patients might over-rely on automated systems), privacy concerns (handling sensitive personal and medical information), and the erosion of trust (incorrect or inconsistent information could erode trust in the reliability of automated technologies). 

One of the most important aspects under scrutiny is the quality of responses that LLMs provide to medical queries including those related to urology and prostate cancer [20]. This line of investigation is well-rooted in studies that seek to address the impact of the Internet as a source of medical information on patients [21]. Cumulating evidence seems to suggest that LLMs have the potential to disrupt and radically reframe the doctor–patient relationship and possibly diminish the asymmetry of knowledge [13]. 

The potential benefits of LLMs should be assessed and validated by healthcare professionals considering the quality of medical information and local cultural specificities. In the context of prostate cancer—a condition that requires detailed and continuous patient education—LLMs can play a crucial role. They can provide comprehensive information about early detection, diagnosis, treatment options, and post-treatment care, all tailored to each patient’s individual needs. Moreover, LLMs can assist in creating personalized health communication strategies that consider a patient’s medical history, preferences, and cultural context. This level of personalization is essential in managing chronic diseases like prostate cancer, where psychological and emotional support is as important as medical treatment. By integrating LLMs into health communication, healthcare providers such as hospitals, outpatient clinics, and family doctors can enhance patient satisfaction, improve adherence to treatment plans, and ultimately achieve better health outcomes.

In 2020, within the European Union’s 27 nations, prostate cancer was responsible for 23.2% of all new cancer cases diagnosed in men excluding non-melanoma skin cancers. Additionally, it accounted for 9.9% of all male cancer deaths, making it the most common cancer among men and the third leading cause of cancer-related deaths, following lung and colorectal cancers [22]. Prostate cancer ranks first in the top five most frequent cancers for males of all ages in Romania [23]. Studies assessing the quality of outputs from LLMs regarding prostate cancer-related questions, as evaluated by medical experts and aimed at optimizing for patient consumption, are still in their nascent stages [24]. Essentially, the extant research can be divided into two threads: one line is focused on how LLMs can be used by patients [25], and the other on how automated solutions can be deployed by medical doctors in the management of medical information [26].

Integrating LLMs into healthcare communication presents significant legal and ethical challenges including concerns about patient privacy, data security, and the accuracy of medical information. LLMs must be designed and deployed in compliance with relevant regulations to prevent data breaches and ensure patient confidentiality. Different countries and regions have varying regulations regarding the protection of patient data (e.g., the General Data Protection Regulation—GDPR—in the European Union and HIPAA in the United States), which establish legal frameworks for collecting, storing, and sharing patient information. In Romania, the regulation of LLMs in healthcare is primarily governed by the GDPR, specifically by the Law of the Citizen’s Right to Personalized Medicine [18]. With the recent approval of the EU AI Act and the European Health Data Space regulation by the European Commission and European Parliament, these will become the main legal frameworks governing the implementation of LLMs in all Member States starting January 2026.

The EU AI Act aims to regulate AI technologies including LLMs to ensure they adhere to high safety standards, transparency, and ethical accountability. This Act categorizes AI applications into different risk levels, requiring stringent compliance for high-risk applications that include healthcare communication tools. Healthcare providers must determine where the responsibility for potential errors in LLM-generated information lies—whether with the technology developers, healthcare institutions, or individual practitioners. Addressing these legal challenges is essential for the safe and effective implementation of LLMs in healthcare communication. This ensures that these technologies enhance, rather than compromise, patient care and trust and facilitate doctor–patient communication. 

Our work builds upon the recently coalescing literature that assesses the quality of LLM responses to queries about prostate cancer for patient consumption [13,27,28]. However, there is a critical need to expand the current literature to include various cultural contexts. Differences in medical practices, patient communication preferences, and health literacy levels across countries and cultures could significantly influence the effectiveness of LLMs. By incorporating studies from diverse geographical and cultural backgrounds, researchers can develop more universally applicable and culturally sensitive AI tools that better address the global disparities in healthcare outcomes. Furthermore, it is interesting to note that existing studies on the performance of LLMs in responding to health-related queries have largely overlooked the cultural dimension. This oversight is surprising, especially considering that OpenAI (San Francisco, CA, USA) acknowledges ChatGPT’s cultural bias toward Western views and its optimal performance in English; therefore, users are advised to carefully consider its content [29]. For instance, research on vaccine hesitancy has shown that ChatGPT’s responses can vary significantly across different languages [30]. Moreover, while there is considerable research into the general benefits and challenges of LLMs in healthcare, there is a paucity of studies that have systematically evaluated these models against established medical guidelines and through the lens of experienced medical professionals [25,27].

To cover this gap, our study critically evaluated the performance of three widely available and large language models—ChatGPT (3.5), Gemini (Pro), and Co-Pilot (the free version)—compared to the Romanian Patient’s Guide on prostate cancer [31] within a well-defined cultural environment (i.e., Romania). Inherently, this research explores two pivotal lines. First, it examines whether LLMs operating in the Romanian language are effective compared to the Patients’ Guide, particularly in light of inherent cultural biases. Second, it provides the opportunity to reflect on the potential impact of integrating LLMs into Romanian medical practice, which could fundamentally transform doctor–patient communication. Our study is not a technical evaluation but rather an exploration of the potential use of large language models (LLMs) in the context of prostate cancer and within various cultural settings such as Romania. Thus, our work may inform the subsequent fine-tuning efforts of LLMs tailored to the Romanian cultural context. 

We hope that our findings may contribute to developing a new conceptual model for doctor–patient communication in the era of LLMs concerning prostate cancer. Moreover, we aim to enrich the discourse on the cultural dimensions of AI usage in healthcare by sharing our findings on the performance of LLMs in addressing prostate cancer-related queries within the Romanian context. This aspect has gained relevance as existing research has demonstrated that while ChatGPT and other LLMs are significant in the global healthcare landscape, language bias remains a key concern [32]. Last but not least, our study adheres to the stream of research that systematically evaluates LLMs in the context of established national patient guides. 

## 2. Materials and Methods

### 2.1. Study Design 

The study’s approach was devised to systematically evaluate the effectiveness of three prominent language models (LLMs: Co-Pilot, the free version, ChatGPT 3.5, and Gemini Pro) in comparison to the official Romanian Patient’s Guide on prostate cancer (i.e., the Guide). To our knowledge, the information about whether the Guide was used to train these LLMs is not publicly available. The official Romanian Guide is typically recommended by medical doctors to their patients and families after the initial consultation and diagnosis of prostate cancer, serving as a detailed and reliable source of information about the disease. Given the increasing number of prostate cancer diagnoses each year and the fact that Romania has one of the lowest numbers of doctors in Europe due to brain drain, the time allocated by medical doctors for patient consultations is often insufficient, sometimes being less than 15 min. In selecting the LLMs, we accounted for their widespread, albeit undocumented, popularity in Romania rather than their technical features. This approach allowed us to analyze the LLMs most representative of those currently used by doctors and laypeople in the Romanian context. By focusing on LLMs that are already popular among Romanian users, we could explore user behavior and model applicability, which complements the technical evaluations found in the broader research landscape. This is in line with our aim to bridge the gap between technical capability and user engagement within specific cultural and linguistic environments. It is worth mentioning here that similar studies used only ChatGPT 3.5 [20,24,25,27,28], different versions of ChatGPT (versions 3.5 and 4.0), or ChatGPT in combination with highly specialized or simply open access LLMs such as Mistral-7B-Instruct-v0.2, Mixtral-8x7B-v0.1, Llama-2-13b-chat, Nous-Hermes-Llama2-70b, openchat-3.5-1210, BioMistral-7B DARE, NeevaAI, and Chatsonic [13,33]. The evaluation focused on the models’ ability to deliver accurate, up to date, comprehensive, and user-friendly information on prostate cancer. To enhance research topic-wise comparability, we decided to deploy similar evaluation criteria previously deployed in analogous studies (i.e., focused on prostate cancer) [20,24,25,27,28,33]. Namely, we dubbed these four criteria under the following labels: *accuracy, timeliness, comprehensiveness,* and being *friendly.* In our design, *accuracy* refers to the correctness of the information provided by the LLMs (i.e., accurate responses should be free from errors and align with verified medical data and guidelines), *timeliness* refers to the promptness with which the LLMs provide information and the extent to which this information is current and up-to-date, particularly crucial in the rapidly evolving field of medical science, *comprehensiveness* measures the depth and breadth of the information provided (i.e., in the context of prostate cancer, this would reflect the ability to cover a range of topics from symptoms and diagnosis to treatment options and possible side effects comprehensively), and *friendly* (or *user-friendliness*) assesses how easy it is for users (both laypeople and medical professionals) to interact with the LLMs (i.e., this includes the ease of understanding the responses, which can significantly impact the effectiveness of communication and subsequent patient outcomes). These distinct criteria align with those used in similar studies and address the key aspects of LLM functionality that are most relevant to users in real-world scenarios. By contextualizing the evaluation within the framework of existing literature, our study contributes to a nuanced understanding of how AI tools like LLMs can support healthcare delivery in culturally specific settings. This approach not only validates the relevance of the chosen evaluation criteria, but also positions our study as a valuable contribution to ongoing discussions about the integration of AI in healthcare, especially in areas with distinct cultural and language considerations. Furthermore, we note here that the evaluation was performed only by medical doctors (these were the participants in our research). We operated under the assumption that, given their ongoing interaction with patients during their medical practice, they could assess the friendly feature of the responses on behalf of laypeople. This approach is convergent with other similar studies in the field [13]. 

Consequently, we produced a set of 25 inquiries that encompassed frequently asked issues concerning prostate cancer. For replication purposes, the code and the data are freely available [34]. The subsequent prompt was employed to query the three LLMs: *I am a man, and my doctor has informed me that I have been diagnosed with prostate cancer. I am interested in learning more about the diagnosis, treatment, and overall management of the disease, which will help me better manage the condition and improve my quality of life. Therefore, I have the following questions for which I would like to obtain answers*. One operator performed queries on all three models to maintain uniformity in collecting the data. The questions were performed in incognito mode on Google Chrome to prevent any customized search biases, guaranteeing that each LLM answered simply based on their expertise and algorithms. Upon gathering the responses, we used a randomized procedure to fully blend the answers. This process aimed to guarantee that the future expert examination would be devoid of any prior biases regarding the origin of each response. 

It is important to recognize that inherent variability in large language models (LLMs) can result in different answers to repeated prompts. In our study, we chose not to prompt the LLMs multiple times as our primary focus was on assessing the overall adequacy of their responses to prostate cancer-related queries rather than the consistency of these responses. Additionally, we aimed to simulate a real-world user experience as closely as possible, where it is less common for users to repeatedly issue the same prompt when seeking medical information.

### 2.2. Participants and Setting

The randomized responses were thereafter given to a panel of eight prostate cancer specialists, specifically medical professionals. These doctors were affiliated with a hospital in Bucharest that treats the highest number of prostate cancer patients annually of any Romanian hospital. We primarily selected this hospital for our study due to its significant volume of prostate cancer cases. We dispatched invitations to all of the medical practitioners (eight medical doctors) specializing in the treatment of prostate cancer who were associated with this particular institution in question. All eight experts agreed to participate in the study. These experts were males with an average age of 38.25 years with a standard deviation of 7.13 and a range of 20. Additionally, they had an average number of 16.88 patients per month with a standard deviation of 25.84 and a range of 79. It was observed that the specialists had a relatively small to moderate range of ages, with a coefficient of variation of 18.63%. However, there was a significant fluctuation in the number of cancer patients treated every month (coefficient of variation: 153.11%), indicating an extremely skewed distribution. The study participants gave their ratings independently. However, before starting the rating exercise, we organized a plenary meeting with the medical doctors during which the evaluation criteria (*accuracy, timeliness, comprehensiveness,* and *friendliness*) were discussed in detail. The rationale of this meeting was to ensure that the eight participants would give valid scores to each response (i.e., standardization of evaluation criteria). Specifically, holding a plenary meeting to discuss the evaluation criteria in detail helped ensure that all participants clearly and uniformly understood what each criterion meant, which is crucial for minimizing subjective variance in their ratings. Beyond discussing the criteria, during the plenary meeting, the participants underwent a simulation exercise using example ratings to ensure consistency. The fact that the participants provided their ratings independently [27] after the calibration discussion illustrates that the evaluation was conducted without peer influence, thereby maintaining the integrity of individual assessments. 

### 2.3. Variables and Procedure 

We devised a data-collecting form that included the replies obtained from each information source (the three LLMs and the Romanian official Patient’s Guide) for each of the 25 relevant questions on prostate cancer. Essentially, there were a total of 100 replies. We requested that each of the eight medical practitioners evaluated these 100 replies based on four criteria: accuracy, timeliness, comprehensiveness, and friendliness. Essentially, each expert assigned a numerical rating of 1 to 5 to each response. Specifically, each of the 100 replies was evaluated based on four criteria: accuracy, timeliness, comprehensiveness, and user-friendliness. 

To preserve the integrity of the evaluation process, the experts were deliberately unaware of the source of each response. In addition, we developed this approach to mitigate the disparities and potential biases that may have resulted from the differences in age and patient volume among the medical practitioners in cancer treatment. Nevertheless, it is crucial to consider the findings with caution due to the homogeneity of the sample in terms of sex assigned at birth as all panel members were male medical practitioners. Given the limited and growing literature on this topic, no existing studies have currently examined the impact of sex on the distribution of responses. However, there may probably be biases in the responses regarding this socio-demographic component, particularly sex.

Each panel member was given a digital copy of the questionnaire. We combined all of the replies into a data frame and performed statistical analysis using the available in RStudio (R 3.6.0+). The data and code are freely available for replication and secondary analysis [34]. All participants willingly accepted taking part in the study after receiving a consent form that contained details on the research goals and related contexts. Additionally, it stressed that the identity of the participants would be kept anonymous and that their participation will be handled with the highest level of confidentiality. Moreover, it underscored that participating in the research was completely optional. The research participants did not receive any incentives, either in the form of money or non-monetary rewards. Nevertheless, we promise to provided them with access to the data frame and all scientific outputs including research reports, scientific publications, oral presentations, and other materials derived from the collected data. Ethics committee approval was not required since no patient data were recorded in this study.

The methods used were conducted under the applicable national and international standards and laws. All subjects provided informed consent. The privacy rights of the experts were respected.

The evaluation procedure was carried out entirely in Romanian, allowing for a seamless comprehension among the native expert panel and enabling an examination of the LLMs’ proficiency in handling and reflecting local and cultural subtleties in their replies. 

### 2.4. Statistical Analysis

We employed various statistical techniques that we thought were suitable for achieving the objectives of our study. Our focus was to determine the extent of variation in ratings based on the evaluation criteria and the extent of heterogeneity among experts in their assessments. 

We computed the mean rating scores for each information tool while accounting for the four assessment criteria (accuracy, timeliness, comprehensiveness, and being friendly). The corresponding descriptive statistics are useful for detecting variation in the assessment scores. Then, we performed an analysis of variance (ANOVA) to examine the differences in scores provided by the experts across the information tools (ChatGPT, the Guide, Co-Pilot, and Gemini) and the four criteria of assessment (accuracy, timeliness, comprehensive, and friendliness). We deployed ANOVA for several reasons. First, we had multiple groups (tools and criteria) and we wanted to determine whether there were statistically significant differences in scores across these groups. Second, by including an interaction term (tools and criteria), we sought to identify the main effects (tools and criteria) and the interaction effects between these factors. The expert scores represented the dependent variable in our ANOVA model. Our main effects were also represented by the differences in mean scores across tools (*tool* effect) and criteria (*criterion* effect). The interaction effect tests whether the influence of a tool depends on the criterion used. An ANOVA model was set to indicate whether both tools and criteria significantly affected the experts’ scores and if the tool’s performance depended on the criterion. Moreover, we used a multivariate analysis of variance (MANOVA) to examine the effect of tools on multiple evaluation criteria simultaneously. In our MANOVA model, we used the scores across the four evaluation criteria as the dependent variable and the information tools as the independent variable. We were interested in gauging how much variation in the evaluation was due to the difference between tools.

We reported the analysis of variance for the tool and criterion effects on the scores. Namely, we compared means across different tools (sources of information) and criteria. We examined how the choice of tools affected the scores given by panel experts, regardless of the criteria (see *tool*). Furthermore, we looked at how the criterion affected the scores, irrespective of the tool (see *criterion*). Aside from these main effects, we assessed the interaction between *tool* and *criterion*: whether the effect of a tool on the scores changed with different criteria. For example, one tool might be better for *accuracy* but not as good for ease of use (i.e., being friendly).

We assessed the reliability and agreement of ratings among the eight experts using Intraclass Correlation Coefficients (ICC). We wanted to determine whether the experts were consistent in scoring and agreed on the absolute scores assigned. We analyzed both the reliability of individual experts and the reliability of the average scores of all experts. Essentially, we aimed to understand the degree of agreement among experts. The ICCs were computed under assumptions of absolute agreement and consistency considering both the individual and average ratings. 

Finally, we fit a linear mixed-effects model to analyze the effects of experts, criteria, and tools on the scores. We addressed the dataset as having a hierarchical structure, where scores were nested within experts, criteria, and tools. We looked at random effects (experts, criteria, and tools) to account for variability among the experts and among the criteria. We also examined the fixed effects to understand the impact of tools on the scores. In our case, the repeated measures represented the situation where each expert provided multiple ratings across different tools and criteria (this required accounting for within-subject correlations). 

In a nutshell, we consider that these statistical analyses provided robust evidence of the significant and variable impact of tools on the evaluation scores across different criteria while also highlighting whether the experts assessed these tools in a consistent and reliable manner when considered as a group.

## 3. Results

Table 1 reports how the panel experts rated each information tool across the four criteria (accuracy, comprehensiveness, timeliness, and being friendly). ChatGPT seems to show, on average, better scores than the other LLMs and the official Guide. We also showcase that the ratings associated with the Guide tend to have a higher variation (spread) than the other tools (i.e., the standard deviation, SD, was higher than 1.1). 

Table 2 reveals the significant main effects of the tool on the evaluation scores (F(3, 3184) = 58.73, *p* < 0.0001) and the evaluation criterion (F(3, 3184) = 35.24, *p* < 0.0001), indicating substantial differences in the scores across different tools and criteria used. Furthermore, the interaction between tool type and evaluation criterion was also significant (F(9, 3184) = 3.35, *p* = 0.0004), suggesting that the impact of each tool on the evaluation scores varied depending on the specific criterion applied. In a nutshell, the statistical analysis was conducted using a two-way ANOVA, which demonstrated significant effects for both the tool and criterion as well as their interaction. 

In the analysis of evaluation scores across multiple criteria (see Table 3), a multivariate analysis of variance (MANOVA) was conducted to determine the effect of the tools on the combined dependent variables: accuracy, comprehensiveness, timeliness, and friendliness. The MANOVA results, as indicated by Pillai’s trace, showed a significant multivariate effect of the tool on the set of evaluation criteria (Pillai’s trace = 0.14811, F(12, 2385) = 10.322, *p* < 0.0001). This significant finding suggests that the differences in tools were not only statistically significant, but also practically relevant across the combined criteria. These results underscore the impact that tool choice has on the evaluation outcomes, confirming that the selection of tools influences various aspects of performance assessment in a measurable and substantial way.

In assessing the consistency of expert ratings, we computed the intraclass correlation coefficients (ICCs) to evaluate the reliability across different assumptions of rater effects. The reliability of individual raters was relatively low, with ICC values around 0.24 to 0.29, suggesting notable variability in individual assessments (F(99, 3069) = 14, *p* < 0.000). However, when averaging across the 32 ratings (i.e., each expert assessed four criteria), the reliability markedly improved, with ICC values ranging from 0.91 to 0.93, indicating the excellent consistency of the averaged ratings (F(99, 3100) = 11, *p* < 0.000) (see Table 4 for detailed ICC statistics)

Table 5 displays a linear mixed-effects model that we fitted to evaluate the impact of the individual experts, criteria, and tools on the evaluation scores (REML criterion = 8801.3). The analysis revealed significant variability among the experts (σ^2^ = 0.13773), with less variability attributed to the criteria and tools. Put differently, differences among the experts accounted for more variability in the scores than the criterion or tool alone. The overall average score was significantly different from zero (intercept = 3.8425, SE = 0.2161, *p* < 0.00001), underscoring the influence of these factors on the score outcomes (random effects: σ^2^_id = 0.3711, σ^2^_criterion = 0.2087, σ^2^_tool = 0.2707; residual σ^2^ = 0.9486). All in all, the results reported in Table 5 suggest significant variability in the scores attributed to differences among experts, with lesser but still notable variability due to the criteria and tools. The overall mean score (intercept) was significantly different from zero, indicating a meaningful average effect across all groups when controlling for random variations.

## 4. Discussion

The results of our study offer useful insights into the assessment of the performance of different LLMs in responding to queries related to prostate cancer by a group of Romanian experts based on various criteria. The ANOVA analysis revealed significant main effects for both the tools and criteria as well as a significant interaction between the tools and criteria. This suggests that the performance of the tools varied depending on the assessment criterion. The MANOVA analysis provided additional evidence, demonstrating an important multivariate effect of the tools on the overall assessment criteria. Intraclass correlation coefficients (ICCs) revealed low reliability among individual experts (ICC = 0.24–0.29), suggesting that individual raters had variable scoring tendencies. However, the high ICCs for the aggregated scores (ICC = 0.91–0.93) indicated that the expert panel, as a whole, provided consistent and reliable evaluations. The linear mixed-effects model provided further insights into the evaluation process. There was significant variability in the baseline scores across experts (variance = 0.13773), criteria (variance = 0.04354), and tools (variance = 0.07331). Despite this variability, the significant overall intercept (estimate = 3.8425, *p* < 0.00001) indicated a consistent average scoring pattern across all experts. In summary, these results demonstrate that while individual experts varied in their scoring tendencies, the overall evaluations were consistent and reliable when considered as a group. The findings also highlight that the performance of different LLMs varied significantly across the evaluation criteria, emphasizing the importance of a multidimensional assessment approach when comparing such tools. In the context of prostate cancer, where accurate, precise, and empathetic communication is critical, LLMs have the potential to play a transformative role [35]. 

The research objective of this paper was to critically evaluate the performance of three widely available LLMs—ChatGPT (3.5), Gemini (Pro), and Co-Pilot (the free version)—compared to the Romanian Patient’s Guide on prostate cancer, in the Romanian context. Our findings suggest that ChatGPT seems to have had, on average, better scores than the other LLMs and the official Patients’ Guide. Nevertheless, this result should be contextualized to the current stage of knowledge and taken with a grain of salt. As a general remark, we should underline that the investigation of the effectiveness (or quality) of LLMs in providing responses to queries related to cancer prostate topics is still at a nascent phase. Research on this topic is rapidly cumulating and coalescing. However, only a critical mass of research that is yet to come will ascertain the degree to which the reported results of evaluating LLMs (e.g., ChatGPT) are a methodological artifact, are affected by the inherent cultural bias, are dependent upon the used language, or by the content of the referenced national and European Guides. For instance, it has already been established as common practice to test the accuracy of responses given by LLMs to various medical questions [13,24,25,27,33,36]. Quality assessment strategies vary from using questions found in standardized medical tests such as the United States Medical Licensing Exam (USMLE) [36] to deriving questions from Google Trends queries [24] or medical guides used as benchmarks [25,27] and evaluating the answers using medical experts. All of the aforementioned studies gave ChatGPT special attention, given its high notoriety status.

Studies akin to ours, which used official patient guides to construct their question banks on prostate cancer and tested responses from ChatGPT through expert evaluation, often referenced the European Urology Association’s (EAU) prostate cancer guidelines [25,27]. These studies reported that ChatGPT demonstrated medium-level performance in accurately responding to the proposed questions. However, several key differences distinguish our study from these efforts. Firstly, our question bank was constructed using the official Romanian Patient’s Guide rather than the EAU guidelines. Secondly, unlike other studies that assumed the accuracy, timeliness, comprehensiveness, and ease of use of their guides as standards, our study tested these attributes against the responses provided by the three LLMs, employing a blinding and randomization procedure. Notably, some researchers [25] classified responses as correct (“true positive”) only if the answer provided by ChatGPT aligned with the EAU guide, while answers deemed correct but not found in the guide were classified as “false positive”, contributing to a higher inaccuracy score. In contrast, our approach did not automatically deem non-guideline responses as inaccurate. Thirdly, the assessment scales used to evaluate the quality criteria differed; for example, Lombardo and colleagues [27] used a four-point scale ranging from “completely correct” to “completely incorrect” for their 195 questions/recommendations. Conversely, our study utilized a five-point Likert scale from ‘poor’ (1) to ‘excellent’ (5) based on specific criteria such as accuracy, timeliness, comprehensiveness, and friendliness (easy to use). These methodological variations including the use of different patient guides and assessment metrics underscore potential avenues for future research to determine whether discrepancies in the results stem from methodological contrasts or from a disparity in the quality of the Romanian guide compared to the EAU guide, which might necessitate a re-evaluation of the Romanian guide. 

From another angle, our results may have several implications. For instance, these can contribute to orienting the contemporary design of the doctor–patient relationship and the transition toward a truly personalized doctor–patient communication model: delivering the right message from the right doctor, at the right time, to the right patient [37,38]. There are numerous ways in which the doctor–patient relationship can be redefined in the context of the current research on the quality of LLMs in assisting with responses to health queries. Our findings may support the idea that tool selection should be targeted based on the intended application. Furthermore, it may be reasonable to expect that aggregating ratings across experts provides a more reliable evaluation of language models. Additionally, future evaluations should consider different weighting schemes for criteria based on their relative importance in specific applications. For brevity, our study is an invitation to reflect on the personalization of doctor–patient communication, taking into consideration the specificities of the information source (the medical doctor), the message, and the medium (LLMs including the Guide). The Patient’s Guide can benefit from the development of LLMs and can transform itself into a dynamic, living, and interactive guide, an essential tool in the new paradigm of personalized communication [39].

In the doctor–patient relationship [40], adapted from the linear model of communication, the interaction begins with the doctor or the patient serving as the information source, depending on who initiates the communication. The doctor may encode complex medical information into understandable language, or the patient may describe symptoms or concerns. The message is then transmitted through channels like face-to-face conversations, phone calls, or digital communication in telemedicine. Once received, the message is decoded by the listener, be it the patient interpreting medical advice or the doctor understanding the patient’s symptoms. Throughout this process, potential noise such as medical jargon, emotional distress, or environmental distractions can interfere with the clarity and effectiveness of the communication. This model illustrates the need for clear and precise dialogue, with careful consideration of the communication medium and potential barriers, to ensure that both the doctor and patient accurately exchange and understand vital health information [41].

**Figure 1:**
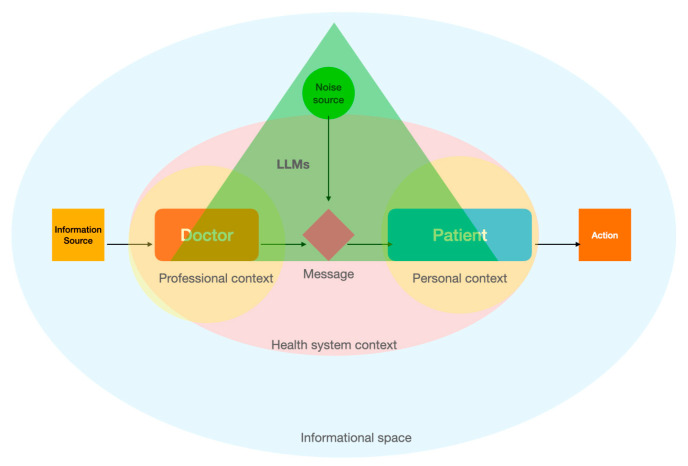
Conceptualization of the personalized communication model through the integration of LLMs in the Shannon–Weaver mathematical model of communication.

Our study contributes to the current efforts to analyze the role of LLMs in accurately informing patients with prostate cancer. One particular important implication refers to the democratization of medical knowledge. We advocate that LLMs could be used by the patients not only independently, but also in tandem with medical doctors (across the entire spectrum of the disease, from prevention to the most sophisticated treatments). In effect, LLMs can become an important part of the dialogue between doctors and patients. By focusing on personalizing communication in the doctor–patient relationship—ensuring that the right message from the right doctor reaches the right patient at the right time—we support the complementary use of LLMs within the doctor–patient dyad, considering their potential to both enhance and complicate medical communication (Figure 1).

Based on their performance, LLMs can play a nuanced role [42] in the linear model of communication between the doctor and patient. LLMs can assist doctors by helping to encode complex oncological information into more accessible language, potentially simplifying explanations about treatment options, side effects, and prognostic outcomes. This assistance can minimize the noise created by medical jargon, making it easier for patients to understand their condition and treatment choices. This would actually be one of the directions in which LLMs can be deployed [26], supplementary to the democratization of medical knowledge to laypeople. 

LLMs might also introduce new forms of noise or interference [43]. For instance, controlling for other factors (cultural bias, repeated prompts, medical topic, etc.), the potential for misinterpretation or oversimplification of medical advice through automated language processing could lead to inaccuracies in the information received by the patient. Additionally, the reliance on technology for communication could inadvertently distance the personal interaction between the doctor and patient, potentially leading to a loss of vital nuances that are often conveyed through direct human contact. Moreover, the impersonal nature of automated interactions could diminish the empathetic communication that is crucial in oncology, where understanding patient fears and emotional needs is as important as discussing clinical treatment.

Reflecting on the idea of the democratization of medical knowledge, the accuracy of automated translations of medical content can sometimes be inconsistent, leading to potential misinterpretations or oversimplifications of critical health information. If not closely monitored, this could lead to a patient misunderstanding the severity of their condition, the expected outcomes of a treatment, or the importance of follow-up care. Therefore, while LLMs have the potential to enhance clarity and understanding in communicating complex medical information, they need careful integration to maintain the essential human connection and trust in the doctor–patient relationship, especially in sensitive areas such as prostate cancer [44,45]. 

There are some limitations that readers should account for. Future studies could include a larger and more diverse panel of experts to improve the generalizability. The inclusion of additional LLMs and criteria could also offer a more comprehensive comparison. Further assessing the impact of cultural bias [29] (e.g., comparing multiple languages [32]) on the performance of LLMs may provide useful insights for their development. Current work has evaluated only one-shot interactions with LLMs. Future work may address the longitudinal evolution of doctor–patient relationships and evaluate how users change their behavior in interrogating the LLMs given this relationship. Relational event models and their extensions [46,47,48] allow for modeling time-ordered interaction events. This approach can integrate both human attributes (doctors and patients) and their personal networks. In effect, it can uncover complex patterns and dynamics, enabling the optimal integration of LLMs into medical practices as well as in social contexts. Also, we acknowledge that our methodology did not account for the variability in responses provided by an LLM to repeated prompts. While this is indeed a valid concern, this was outside the scope of our research objective. Nonetheless, this aspect certainly warrants further investigation in future studies. Additionally, we developed our research design based on similar studies in the field [25,27]. We operated under the assumption that, given their daily interactions with patients, the medical doctors in our study possessed the necessary capacity to assess the friendliness of the LLMs’ responses. Future work may extend to include patient assessments of LLM responses on this criterion and examine potential variations in ratings. Moreover, akin to other similar studies, we did not perform any fine-tuning [49] on the LLMs, with the risk of the LLMs’ performance not being at their best. Last but not least, assessing tool performance over time may provide information on the adaptability and learning abilities of the different language models. 

## 5. Conclusions

In this paper, we examined whether LLMs operating in the Romanian language were effective compared to the Patients’ Guide, particularly in light of inherent cultural biases. As reported, the results are seemingly promising. We also took the opportunity to reflect on the potential impact of integrating LLMs into Romanian medical practice, which could fundamentally transform doctor–patient communication. In this context, we referred to the possible democratization of medical knowledge as well as the facilitative and disruptive elements that LLMs may have on the doctor–patient relationship. Our results may have implications for reframing the doctor–patient relationship and transitioning toward a possible personalized health communication model: delivering the right message from the right doctor at the right time to the right patient. For example, this may have a particular application in the management of prostate cancer. In Romania, cancer patients expect to receive information about their disease primarily from their doctors, who are considered the most trusted source [50]. Digital tools serve as the second most important source of information. Our study provides insights into the quality of the responses generated by LLMs on the topic of prostate cancer in comparison to the current standard, the Patients’ Guide. This is particularly important in the context of a shortage of healthcare professionals and an increased number of hospitalized patients due to the rising prevalence of prostate cancer in Romania.

## Figures and Tables

**Table 1:** Descriptive statistics.

	Tool	Criterion	Mean	Median	SD
1	ChatGPT	Accuracy	4.13	4	0.816
2	ChatGPT	Comprehensiveness	4.08	4	0.772
3	ChatGPT	Timeliness	4.15	4	0.831
4	ChatGPT	Friendly	4.30	4	0.796
5	Guide	Accuracy	3.51	4	1.340
6	Guide	Comprehensiveness	3.26	3	1.370
7	Guide	Timeliness	3.86	4	1.100
8	Guide	Friendly	3.83	4	1.190
9	Gemini	Accuracy	3.51	4	1.010
10	Gemini	Comprehensiveness	3.22	3	0.932
11	Gemini	Timeliness	3.74	4	0.913
12	Gemini	Friendly	3.99	4	0.924
13	Co-Pilot	Accuracy	3.84	4	1.100
14	Co-Pilot	Comprehensiveness	3.84	4	0.983
15	Co-Pilot	Timeliness	3.98	4	0.929
16	Co-Pilot	Friendly	4.22	4	0.847

**Table 2:** Analysis of variance (ANOVA) for tool and criterion effects on scores.

	Df	Sum Sq	Mean Sq	F Value	*p*-Value
*Main effects*					
Tool	3	179	59.54	58.73	<0.000
Criterion	3	107	35.73	35.24	<0.000
*Interaction effect*					
Tool:Criterion	9	31	3.40	3.35	0.0004
Residuals	3184	3228	1.01		

**Table 3:** Multivariate analysis of variance (MANOVA) results for tool impact on evaluation criteria.

Source	Pillai’s Trace	Approx. F-Value	Df	Numerator Df	Denominator Df	*p*-Value
Tool	0.14811	10.322	3	12	2385	<0.001
Residuals			769			

Note: *p* < 0.001, indicating a statistically significant multivariate effect of tools on the evaluation criteria.

**Table 4:** Intraclass correlation coefficients (ICCs) for rater reliability assessment.

	ICC	F	Df1	Df2	*p*-Value
Single raters absolute	0.24	11	99	3100	<0.000
Single random raters	0.24	14	99	3069	<0.000
Single fixed raters	0.29	14	99	3069	<0.000
Average raters absolute	0.91	11	99	3100	<0.000
Average random raters	0.91	14	99	3069	<0.000
Average fixed raters	0.93	14	99	3069	<0.000

**Table 5:** Summary of the linear mixed-effects model for scores by experts, criteria, and tools.

Model Fit Statistics					
Number of observations	3200				
REML Criterion	8801.3				
Random Effects	Variance	Std. Dev.			
id (Intercept)	0.13773	0.3711			
criterion (Intercept)	0.04354	0.2087			
tool (Intercept)	0.07331	0.2707			
Residual	0.89983	0.9486			
Fixed effects	Estimate	Std. Error	Df	t-value	*p*-value
Intercept	3.8425	0.2161	10.8697	17.78	<0.00001

## Data Availability

The data, the questionnaire, the code, and other related files are freely available for replication and secondary data analysis at https://doi.org/10.5281/zenodo.11217682 (accessed on 16 July 2024).
